# Antityphoid and radical scavenging properties of the methanol extracts and compounds from the aerial part of *Paullinia pinnata*

**DOI:** 10.1186/2193-1801-3-302

**Published:** 2014-06-23

**Authors:** Paul Keilah Lunga, Jean de Dieu Tamokou, Simeon PC Fodouop, Jules-Roger Kuiate, Joseph Tchoumboue, Donatien Gatsing

**Affiliations:** Laboratory of Microbiology and Antimicrobial Substances, Faculty of Science, University of Dschang, P.O. Box 67, Dschang, Cameroon; Laboratory of Animal Physiology and Health, FASA, University of Dschang, P.O. Box 222, Dschang, Cameroon; Laboratory of Phytobiochemistry and Medicinal Plants Study, Faculty of Science, University of Yaoundé 1, P.O. Box 812, Yaoundé, Cameroon

**Keywords:** *Paullinia pinnata*, Methylinositol, Oleanane-type triterpenoids, Antityphoid, Radical scavenging properties

## Abstract

*Paullinia pinnata* Linn (Sapindaceae) is a medicinal plant, locally used in the West Region of Cameroon for the treatment of typhoid fever. This work was designed to evaluate the antityphoid and antioxidant activities of the extracts and compounds of *P. pinnata*.

The methanol extracts of the leaves and stems were tested for antityphoid and antioxidant activities. Compounds were isolated, and their structures elucidated by analysis of spectroscopic data in conjuction with literature data and tested for the same activities. The leaf extract was also tested *in vivo* for its antityphoid potential in a *Salmonella typhimurium*-induced typhoid fever model in *Wistar* rats.

Seven known compounds: methylinositol (1), β-sitosterol (2), friedelin (3), 3β-(β-D-Glucopyranosyloxy)stigmast-5-ene (4), (3*β*)-3-*O*-(2′-Acetamido-2′-deoxy-*β*-D-glucopyranosyl) oleanolic acid (5), (3*β*,16*α-*hydroxy)-3-*O*-(2′-Acetamido-2′-deoxy-*β*-D-glucopyranosyl) echinocystic acid (6) and (3*β*,)-3-O-[β-D-glucopyranosyl-(1″-3′)-2′-acetamido-2′-deoxy-β-D-galactopyranosyl]oleanolic acid (7) were isolated. Compounds 5 and 1 showed the highest antibacterial (MIC = 0.781-1.562 μg/ml) and DPPH radical scavenging (RSa50 = 19.27 ± 4.43 μg/ml) activities respectively. The maximum extract dose (446.00 mg/kg bw) had comparable activity with ciprofloxacin (7.14 mg/kg bw) and oxytetracycline (5 mg/kg bw). The extract induced significant dose-dependent increase of WBCs and lymphocytes.

These results support the ethnomedicinal use of *P. pinnata* and its isolated Compounds could be useful in the standardization of antityphoid phytomedicine from it.

## Background

Salmonella is one of the genera of the Enterobacteriaceae family. Among the Salmonellae of medical importance are *Salmonella typhi, Salmonella paratyphi* A*, Salmonella paratyphi* B; which cause typhoid fever, paratyphoid A and B fevers respectively (Ammah et al. 1999; Gatsing et al. 2006). *Salmonella typhimurium* is the species responsible for typhoid fever in animal experimental models. Worldwide, there is an estimated 16 million episodes of typhoid fever causing 600 000 deaths each year; the overwhelming majority of infections and deaths occurring in developing countries where typhoid fever is endemic (WHO 1996), associated to peritonitis due to the perforation of ulcerated Peyer’s patches within the small intestine (Everest et al. 2001). These intestinal complications are due only to *Salmonella enterica serotype Typhi*. Conventional antimicrobial drugs are becoming more and more unavailable to the common man in Africa due to increased costs (Gatsing et al. 2007a). In addition, there is a greater resistance to all the three first line antimicrobials (i.e. chloramphenicol, ampicillin and co-trimoxazol) (WHO 1992). Moreover, chloramphenicol which for long had been the drug of choice for the treatment of typhoid fever, has been withdrawn from the market due to its medulary toxicity (medulary aplasia) (Nauciel and Vildé 2005).

This infection produces an acute inflammation that make the lymphoid tissues stand out from the surrounding mucosa. Moreso, the entrance of Salmonella into the body causes the production of superoxide and nitric oxide which react together to form peroxynitrite a strong biological oxidant (Rastaldo et al. 2007). Oxidative stress occurs when organisms encounter elevated levels of reactive oxygen species, such as superoxide anion, hydrogen peroxide, and hydroxyl radical. Reactive oxygen intermediates are produced at low rates during aerobic respiration in most cells, including prokaryotic cells. To cope with oxidative stress, bacteria have evolved protective responses that enable them to counter the damage and survive. Thus, if the bacteria are prevented from producing reactive oxygen compounds, it can contribute to efficiently fight against the microorganism. When bacteria interact with a eukaryotic host, large quantities of reactive oxygen intermediates are produced (Kamlesh et al. 2007) by phagocytes during uptake of microorganisms, and this is a major microbicidal effector mechanism against pathogenic bacteria. Reactive oxygen species can lead to serious health problems including sickle cell diseases, artherosclerosis, Parkinson’s disease, heart failure, myocardial infarction, Alzheimer’s disease, Schizophrenia, and chronic fatigue syndrom (De Diego-Otero et al. 2009). On the other hand, antioxidant compounds such as polyphenols, phenolic acids, flavonoids, and carotenoids (Magadula et al. 2011) are thought to prevent chronic complications in part through their interactions with reactive oxygen species (ROS) and their ability to scavenge free radicals (Seifried et al. 2007). Thus, it may be interesting to find a medicinal plant with dual antimicrobial and antioxidant properties.

*Paullinia pinnata* Linn (Sapindaceae) is a liana used in the West Region of Cameroon for the treatment of bacterial infections like typhoid fever, syphilis, gonorrhea, diarrhea and symptoms like stomach-ache and waist pain. In east Africa, the leaves are reported to be used in the treatment of gonorrhea, wounds and microbial infections (Annan et al. 2009). Previous phytochemical investigations have shown the presence of triterpene saponins and cardiotonic catechol tannins (Bowden 1962; Kerharo and Adam 1974), flavone glycosides (Ehab et al. 1999), steroids and steroidal glycosides (Dongo et al. 2009), a cerebroside and a ceramide (Dongo et al. 2009), as well as antibacterial fatty acids (Chabra et al. 1991) in *P. pinnata* collected from different parts of Africa.

This work was aimed at evaluating the effects of methanol extracts and compounds of *P. pinnata in vitro* for antisalmonellal and radical scavenging activities and *in vivo* for antityphoid activity in a *Salmonella typhimurium*-induced typhoid model in rats.

## Methods

### Plant materials

The air-dried leaves and stems of *P. pinnata* were obtained from Dschang, West Region of Cameroon, in January 2009. The identification of plant specimens was done at the Cameroon National Herbarium in Yaounde by Mr Tadjouteu Fulbert, where a voucher specimen was deposited under the reference number 10702/SRFCam.

### Extraction and isolation

The air-dried leaves (2.04 Kg) and stems (2.02 kg) of *P. pinnata* were powdered and extracted with MeOH (7 l × 2, 48 h each) at room temperature to give crude extracts (233.8 g and 152.17 g respectively) after concentration under reduced pressure. The leaf extract (230 g) was exhaustively and successively partitioned with hexane and acetone to afford the hexane (45.2 g), acetone (8 g) and methanol residue (156.8 g) fractions while the stem extract was partitioned into petroleum ether, ethyl acetate and water to obtain the PE fraction (8.08 g), EtOAc fraction (9.13 g) and aqueous residue fraction (109.89 g).

One hundred and fifty grams of the methanol residue fraction was applied to neutral silica gel 60 (0.2-0.5 mm) column (60 × 8 cm) and eluted with mixtures of n-hexane/ethyl acetate and ethyl acetate-methanol of increasing polarity (100:0 → 0:100 with constant polarity increase of 5%) to give 60 fractions which were further grouped on the basis of their TLC band pattern similarities into 5 fractions (F1 to F5). Further column purification of F2 (eluted with EtOAc-MeOH 90:10) on silica gel yielded six fractions denoted F2.1 to F2.6. White niddle-like crystals, formed in F2.3 (EtOAc-MeOH, 95:5) and F2.4 (EtOAc-MeOH, 90:10) were collected and purified on a sephadex gel (LH-20), eluted with an isocratic system of CHCl_3_-MeOH (40:60) to afford methylinositol (28 mg). Fourty grams of the hexane fraction was applied to neutral silica gel 60 (0.2-0.5 mm) column (60 × 8 cm) and eluted with mixtures of petroleum ether-ethyl acetate of increasing polarity (100:0 → 50:50 with constant polarity increase of 5%) to give 40 fractions. These fractions were further grouped on the basis of their TLC band pattern similarities into 5 fractions (F1 to F5). Fraction F1 (Petroleum ether 100%) was mounted on a silica gel column and eluted with a mixture of Hex-AtOAc of increasing polarity (95:5 → 50:50) to yield 30 fractions which were equally grouped on the basis of their TLC band pattern similarities into 5 sub fractions (F1.1 to F1.5). Sub fractions F1.1 (Hex-EtOAc, 90:10) and F1.4 (Hex-EtOAc, 65:35) both yielded white powders which were purified by sephadex gel (LH-20) column chromatography and eluted with CHCl_3_-MeOH (4:6) to afford β-sitosterol (20 mg). Finally, F2 (Petroleum ether-EtOAc, 95:5) was mounted on a silica gel column and eluted with a mixture of Hex-EtOAc of increasing polarity (95:5 → 70:30) to yield 10 fractions which were grouped on the basis of their TLC band pattern similarities into 3 sub fractions (F2.1 to F2.3). Sub fractions F2.1 (Hex-EtOAc, 95:5) and F2.2 (Hex-EtOAc, 93:7) yielded transparent crystals which were purified by sephadex gel (LH-20) column chromatography and eluted with CHCl_3_-MeOH (4:6) to afford friedelin (18 mg).

The EtOAc fraction (7.07 g) was subjected to column chromatography on Rp-18 gel (MPLC, MeOH-H_2_O 50:50 → 100:0) to afford 3β-(β-D-Glucopyranosyloxy)stigmast-5-ene (119 mg), 3-0-(2′-acetamido-2′-deoxy-β-D-glucopyranosyl)olean-12-en-28-oic acid (170 mg) and 8 fractions. Similarly, F4 (3.60 g) was chromatographed on silica gel column and eluted with CHCl_3_-MeOH (9:1 → 7:3) to give 4 fractions. F4.4 (448 mg) was subjected to sephadex LH-20 gel column chromatography and eluted with CHCl_3_-MeOH (1:1) to afford 3-0-(2-acetamido-2′deoxy-β-D-glucopyranosyl)-l6α-hydroxyolean-l2-en-28-oic acid (45 mg). The aqueous residue fraction (76.79 g) was mounted on a D101 macroporous resin column and eluted successively with H_2_O-EtOH (10:0; 7:3; 5:5; 3:7; 0:10) to obtain 5 fractions denoted F1 to F5 respectively. F5 (946 mg) was purified on a silica gel column, eluted with a stepwise gradient mixture of CHCl_3_-MeOH-H_2_O (8:2:0.5 → 6:4:0.5) to afford 3-O-[β-D-glucopyranosyl-(1″-3′)-2′-acetamido-2′-deoxy-β-D-glucopyranosyl]olean-12-en-28-oic acid (40 mg).

### Identification of isolated compounds

Optical rotations were measured with a JASCO P-1020 digital polarimeter. UV spectra were obtained using a Shimadzu UV-2401 PC spectrophotometer. IR spectra were recorded on a Bruker Tensor-27 infrared spectrophotometer using KBr pellets. 1D and 2D NMR spectra were performed on Bruker AM-400 and DRX-500 spectrometers (Bruker BioSpin GmBH, Rheinstetten, Germany) with TMS as the internal standard. ESIMS spectra were recorded on a Bruker HTC/Esquire spectrometer. HREIMS was recorded on a Waters AutoSpec Premier P776 spectrometer. Column Chromatography (CC) was performed on silica gel (200–300 mesh, Qingdao Marine Chemical Ltd., Qingdao, China), Rp-18 (40–63 μm, Merk). Fractions were monitored by TLC (GF254, Qingdao Marine Chemical Ltd., Qingdao, China), and by heating silica gel plates sprayed with 10% H_2_SO_4_ in ethanol. GC analysis was performed on an HP5890 gas chromatograph equipped with a H_2_ flame ionization detector.

## *In vitro*antisalmonellal assays

### Microorganisms

Four *Salmonella* species were used in this study, one strain from the American Type Culture Collection *(Salmonella typhi* ATCC6539) and three clinical isolates *(Salmonella paratyphi* A*; Salmonella paratyphi* B and *Salmonella typhimurium)* from “Centre Pasteur” of Yaounde-Cameroon.

### Preparation of bacterial inocula

The preparation of bacterial inocula was done using 18 h old bacterial cultures prepared in nutrient agar. A few colonies of bacteria were collected aseptically and introduced into 10 ml of sterile 0.90% saline solution. The concentration of the suspension was then standardized by adjusting the optical density to 0.10 at 600 nm, corresponding to bacterial cell suspension of about 10^8^ colony-forming units/ml (CFU/ml) (Tereshuck et al. 1997). This cell suspension was diluted 100 times to obtain 10^6^ CFU/ml for the assay.

### Determination of minimum inhibitory concentration (MIC) and minimum bactericidal concentration (MBC)

The broth micro-dilution method was used for susceptibility testing of bacteria species. The crude extract, its fractions and compounds were tested against the four *Salmonella* species listed above. The tests were carried out in 96-micro well sterile plates as previously described (Newton et al. 2002). For this, the test substances were dissolved in 5% (v/v) Tween 80 solution and serial two-fold dilutions were made with Mueller Hinton broth to yield volumes of 100 μl/well. One hundred microlitres of 10^6^ CFU/ml bacterial suspensions were added to respective wells containing the test samples and mixed thoroughly to give final concentration ranges of 6250–12.20 μg/ml (for extract and fractions) and 100–0.781 μg/ml (for compounds). The dilution solution, 5% Tween 80, did not show inhibitory effects on the growth of the bacteria. The cultured micro plates were covered and incubated at 37°C for 24 h. Inhibitory concentrations of the extracts were detected after addition of 50 μl of 0.20 mg/ml MTT (3-(4,5-Dimethylthiazol-2-yl)-2,5-diphenyltetrazolium bromide, Sigma-Aldrich, South Africa) and incubation at 37°C for 30 min (Mativandlela et al. 2006). Viable bacteria change the yellow dye MTT to a blue color. The lowest concentration at which no visible color change was observed was considered as the MIC. The bactericidal concentrations were determined by adding 50 μl aliquots of the preparations (without MTT), which did not show any visible color change after incubation during MIC assays, into 150 μl of extract-free Mueller Hinton broth. These preparations were further incubated at 37°C for 48 h and bacterial growth was revealed by the addition of MTT as above. The lowest concentration at which no visible color change was observed was considered as the MBC. Gentamycin and Ciprofloxacin were used as reference drugs and tests were performed in triplicate.

### *In vivo*therapeutic test

This test was carried out using a *Salmonella typhimurium*-induced typhoid model in *Whistar* rat. The *Whistar* rats (7–8 weeks and 150–170 g) were reared in the Animal house of the Department of Biochemistry, University of Dschang-Cameroon. Only the crude extract was used in the treatment of infected animals. Prior to the test, animals were housed under the test conditions for a period of one week.

### Ethical guidelines

The experiments were conducted according to the ethical guidelines of Committee for Control and Supervision of Experiments on Animals (Registration no. 173/CPCSEA, dated 28 January, 2000), Government of India, on the use of animals for scientific research.

Animals were immunosuppressed two day before infection by the oral administration of 30 mg/kg bw. of cyclophosphamide as previously described (Abhishek et al. 2008).

### Typhoid induction

A *Salmonella typhimurium* suspension was prepared at 0.5 Mc Farland turbidity scale as above. This solution (1 ml), containing about 10^8^ CFU was orally administered to each animal (Kamgang et al. 2006). Only infected animals were selected on the basis of their fecal colony counts and used.

### Grouping of animals

Animals were arranged into eight groups of four animals each, two males and two females in separate cages according to sex. Except for group 1 animals which were not infected, the rest were infected. The animals were treated as follows:

-  Group one (reference groups) was not infected and received distilled water during the treatment period.
-  Group two (negative control group) received only distilled water during the treatment period.
•Group two (negative control group) received only distilled water during the treatment period.
-  Groups three and four (positive control groups) received ciprofloxacin (7.14 mg/kg bw) and oxytetracycline (5 mg/kg bw) during the treatment respectively.
-  Group five, six, seven and eight (test groups) received the *P. pinnata* leaf extract at concentrations of 55.75, 111.50, 223.00 and 446.00 mg/kg bw, corresponding to MIC, 2MIC, 4MIC and 8MIC respectively.

• Group five, six, seven and eight (test groups) received the *P. pinnata* leaf extract at concentrations of 55.75, 111.50, 223.00 and 446.00 mg/kg bw, corresponding to MIC, 2MIC, 4MIC and 8MIC respectively.

Food and water were given to the animals before and during the treatment *ad libitum*. Treatment was done by administering the extract orally, every morning at the same time. Each day, the fecal matter was collected during the administration process and assessed for the stool bacterial density. The extent to which the animals complied with treatment was studied by counting the amount of bacterial colonies in the fecal samples using the following protocol.

-  0.10 g of fecal matter was completely dissolved in 5 ml of autoclaved distilled water.
-  50 μl of the resulting solution was spread on the surface of solidified 0.9% saline SS agar in the 35 mm type Petri dish.
• 50 μl of the resulting solution was spread on the surface of solidified 0.9% saline SS agar in the 35 mm type Petri dish.
-  After incubation for 18 h at 37°C, the number of colonies following growth of *Salmonella typhimurium* in each Petri dish was counted and recorded.
-  The results were converted into the number of colonies per gram of fecal matter per animal.
• The results were converted into the number of colonies per gram of fecal matter per animal.

### Effect of extract treatment on hematological parameters

Each time a group was completely healed, the animals were sacrificed by chloroform anaesthesia and blood was collected, by cardiac puncture after dissection, into heparinized tubes. Blood parameters including hematocrite, Red Blood Cells (RBCs), White Blood Cells (WBCs), lymphocytes, monocytes, eosinophils, basophils and neutrophils were evaluated using the heparinized blood (Benson and Cales 1992; Theml 2000).

### Radical scavenging activity

The radical scavenging activities of crude extract, fractions and compounds were evaluated spectrophotometrically using the stable 1,1-diphenyl-2-picrylhydrazyl (DPPH) free radical (El-Ghorab et al. 2006). When DPPH reacts with an antioxidant compound, which can donate hydrogen, it is reduced. The changes in color were measured at 517 nm under UV/Visible light spectrophotometer (Jenway, model 1605). Pure methanol was used to calibrate the counter and all test samples were prepared in methanol (4 mg/ml for plant extracts, compounds and vitamin C, the reference drugs). Twofold serial dilutions were made to obtain a concentration range from 2000–62.50 μg/ml. The methanol solution of DPPH (20 mg/l) was prepared daily. The absorbance (Ab) of DPPH without the test samples was read. The mixtures were made by adding 100 μl of test sample to 900 μl of DPPH solution. The content was mixed and incubated at room temperature in the dark. The absorbance (As) was recorded after 30 min. Experiments were carried out in triplicates and the percentages of DPPH reduction by test samples were compared to that of vitamin C (reference drug) and were calculated by the following formula: 

*Where,*

*Ab: absorbance of DPPH solution without test sample,*

*As: absorbance of DPPH solution mixed with the test sample,*

*RSa: radical scavenging activity.*

The radical scavenging percentages were plotted against the logarithmic values of concentration of test samples and a linear regression curve was established in order to calculate the RSa_50_, which is the amount of sample necessary to decrease by 50% the total free radical DPPH (Yassa et al. 2008).

### Statistical analysis

The data were subjected to one-way analysis of variance, and differences between samples at P ≤ 0.05 were determined by Waller-Duncan test using the Statistical Package for the Social Sciences (SPSS) program. The experimental results were expressed (where appropriate) as mean ± standard deviation of three replicates.

## Results and discussion

**Figure 1:**
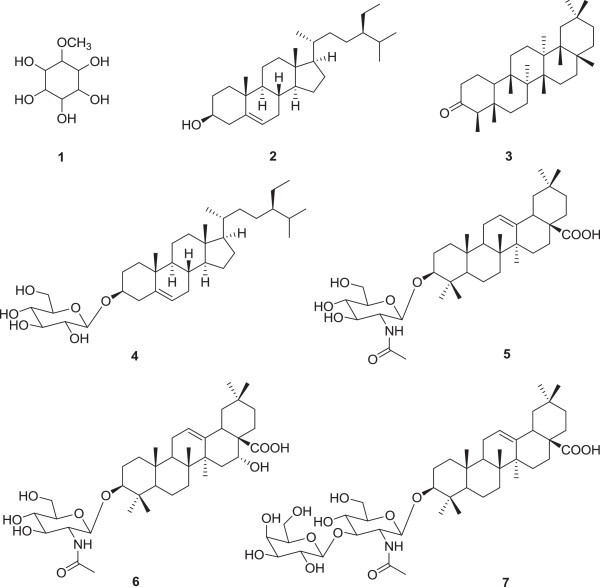
**Chemical structures of compounds from the leaves (1-3) and stems (4-7) of** ***P. pinnata.***

The following known compounds: methylinositol (1) (Zafer et al. 2007) whose ^13^C NMR data were very close to those of L-quebrachitol (Kallio et al. 2009; De Almeida et al. 2012), β-sitosterol (2) (Gupta et al. 2011), friedelin (3) (Klass et al. 1992; Mahato and Kundu 1994) (Figure 1) were isolated and identified in the leaves of *P. pinnata*. From the MeOH stem extract, 3β-(β-D-Glucopyranosyloxy)stigmast-5-ene or daucosterol (4) (Alam et al. 1996), 3-0-(2′-acetamido-2′-deoxy-β-D-glucopyranosyl)olean-12-en-28-oic acid (5) (Ngassapa et al. 1993; Abdel-Kader et al. 2001), 3-0-(2-acetamido-2′deoxy-β-D-glucopyranosyl)-l6α-hydroxyolean-l2-en-28-oic acid (6) (Ngassapa et al. 1993) and 3-O-[β-D-glucopyranosyl-(1″-3′)-2′-acetamido-2′-deoxy-β-D-glucopyranosyl]olean-12-en-28-oic acid (7) (Arafa et al. 2005) were isolated and identified (Figure 1). The compounds isolated in the present study were formerly isolated from other plants and the biological activities of some were demonstrated (Lee et al. 2007; Jiang et al. 2013). *P. pinnata* extract has been proven to possess vascular relaxation properties (Chabra et al. 1991).

The crude extracts and isolated compounds showed variable antibacterial activities against the tested salmonellae species (Table 1). *Salmonella typhi* was less sensitive to all the tested substances. The isolated compounds were less efficient on salmonella than the reference drugs except compound 5 which showed strong antibacterial activity, comparable to ciprofloxacin in some cases. The structures of 5 and 7 are similar, but 5 showed stronger antibacterial activity compared to 7 against the tested species. This suggests that the introduction of a *β*-D-galactopyranose group at C-3*′* of the sugar moiety of C-3 reduced the antisalmonellal activity of 7. In addition, the structure-activity relationship shows that the introduction of an -OH group at C-16 in compound 6 considerably reduced its antibacterial activity. More so, comparing the MIC and MMC values of compound 2 and its analogue 4, it is seen that the presence of the C-3*- β*-D-glucopyranose group in 4 considerably increased the antibacterial activity of the latter. Thus, the presence or absence of C-16-OH and C-3*′-* or C-3 *β*-D-hexopyranose groups play a critical role in reducing or increasing the anti-salmonellal properties of these types of oleanane triterpenoids and steroidal terpenes.

**Table 1:** **Minimum inhibitory (MIC) and bactericidal (MBC) concentrations (μg/ml) of extracts and compounds from** ***P. pinnata***

Test substance	Parameter (μg/ml)	***S. typhi***	***S. paratyphi*** A	***S. paratyphi*** B	***S. typhimurium***
Extract^a^	MIC	781	48	781	781
	MBC	781	390	3125	3125
	MBC/MIC	1	8	4	4
Extract^b^	MIC	781	781	390	390
	MBC	1562	1562	1562	3125
	MBC/MIC	2	2	4	8
1	MIC	6.25	1.562	1.562	25
	MBC	50	12.5	12.50	100
	MBC/MIC	8	8	8	4
2	MIC	100	100	100	100
	MBC	/	/	/	/
	MBC/MIC	nd	nd	nd	nd
3	MIC	25	50	100	100
	MBC	50	100	100	/
	MBC/MIC	2	2	1	nd
4	MIC	50	25	12.5	25
	MBC	100	100	50	50
	MBC/MIC	2	4	4	2
5	MIC	1.562	1.562	0.781	0.781
	MBC	1.562	1.562	1.562	0.781
	MBC/MIC	1	1	2	1
6	MIC	25	25	12.50	12.5
	MBC	25	50	50	25
	MBC/MIC	1	2	4	2
7	MIC	25	50	3.125	12.5
	MBC	50	100	6.250	12.5
	MBC/MIC	2	2	2	1
Ciprofloxacin	MIC	0.195	0.195	1.562	0.781
	MBC	0.781	0.781	1.562	3.125
	MBC/MIC	4	4	1	4
Gentamycin	MIC	6.25	1.562	3.125	3.13
	MBC	12.5	6.25	12.5	6.25
	MBC/MIC	2	4	4	2

Extract^a^: methanol extract of the leaves; Extract^b^: methanol extract of the stems; nd: not determined (greater than 100 μg/ml).

Compound 2 (B-sitosterol) was formally isolated from *Citrus grandis* fruits and shown to possess activity against gram-positive (*Bacillus cereus, Bacillus subtilis* and *Staphilococcus aureus*) and gram-negative (*Escherichia coli* and *Salmonella enteritidis*) bacteria, with MIC value of 300 μg/ml (Matook et al. 2005). Compound 3 (Friedelin), isolated from the stem bark of *Vismia rubescens* demonstrated antibacterial activities against *Salmonella typhi, Staphylococcus aureus, Pseudomonas aeruginosa* with MIC values of 25–200 μg/ml. (Tamokou et al. 2009). The MBC/MIC ratios were generally less than or equal to 4 for extract and most of the compounds, indicating the bactericidal nature of the tested samples on the Salmonellae species (Lalitagauri et al. 2004; Gatsing et al. 2007b). These compounds are isolated from *P. pinnata* for the first time and the antibacterial activities of Compounds 1, 5–7 are being reported herein for the first time.

The crude extract of the leaves was tested *in vivo* on a *Salmonella typhymurium*-induced typhoid model in *Wistar* rats. In infected animals, stools were either soft with mucus, liquid or were moulded and smooth but mucus coated. Sometimes, the presence of blood and mucus made the stool to appear dark and shinny. These animals were weak and less active, with their fur standing at right angles to the body surface instead of the normal sleeping position. The slender body became more bulky, with some of the animals even ‘coughing’. All of these characterized the establishment of infection in the experimental animals, which was clearly revealed by the growth of Salmonella colonies on Petri dishes after the culturing of fecal matter.

**Figure 2:**
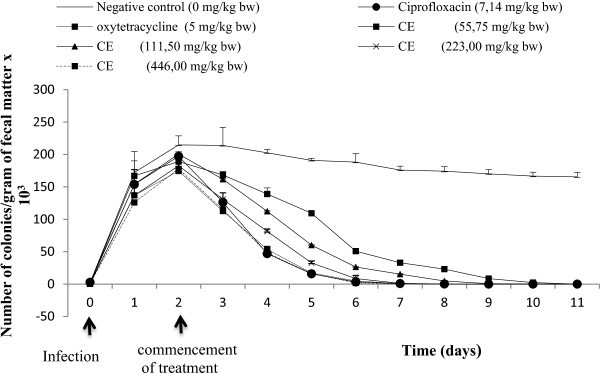
**Evolution of treatment of infected rats as a function of** ***P. pinnata*** **extract dose.** CE: Crude extract.

Treatment with plant extracts improved the general condition of animals. The bacterial load (colonies/gram of fecal matter) significantly (p < 0.05) dropped with time in a dose-dependent manner compared to the negative control (group 2) during treatment (Figure 2). From this Figure, groups 3 (ciprofloxacin), 4 (oxytetracycline), 5 (55.75), 6 (111.50), 7 (223.00) and 8 (446.00 mg/kg bw of extract) were healed after 4, 4, 8, 6, 5 and 4 days of treatment respectively. No significant difference (p ≥ 0.05) was noted in terms of the number of colonies in fecal matter between the extract at the highest dose (446 mg/kg bw) and the reference antibiotics (ciprofloxacin and oxytetracycline) which collectively stopped bacterial multiplication in four days of treatment. In the other treatment groups, the disease condition was eliminated progressively as a function of the extract dose. In the negative control (the untreated group), the stool bacterial charge remained relatively high compared to those of the treated groups throughout the test period. Analysis of blood parameters after treatment shows that, apart from WBCs and lymphocytes which significantly increased (p < 0.05) in a dose-dependent manner in the extract treated groups, the rest of the hematological parameters did not show any significant treatment related variation (Table 2). Classically, during typhoid fever infection, there is leukopenia, a decrease in the number of circulating white blood cells, with eosinopenia and relative lymphocytosis. There is also a positive reaction for *Salmonella typhi* or *paratyphi* on blood cultures (Weinberg et al. 2008). *P. pinnata* crude extract could therefore fight against typhoid fever by inducing the proliferation of WBCs and lymphocytes in the body as revealed in this study.

**Table 2:** **Variation of hematological parameters of rats after treatment with** ***P. pinnata*** **methanol leaf extract**

Blood parameter	Dose (mg/Kg bw.)							
	0	55.75	111.5	223	446	Cipro (7.14)	Oxy (5.00)	Ref
Hematocrite (%)	35.71 ± 4.71^a^	37.78 ± 3.76^a^	33.81 ± 6.240^a^	39.22 ± 2.51^a^	36.04 ± 1.27^a^	37.59 ± 2.13^a^	37.35 ± 2.44^a^	38.36 ± 1.58^a^
RBC Count (10^6^ mm^−3^)	4.023 ± 0.096^a^	4.016 ± 0.090^a^	4.126 ± 0.138^a^	4.030 ± 0.160^a^	4.053 ± 0.070^a^	4.210 ± 0.253^a^	4.193 ± 0.209^a^	4.333 ± 0.145^a^
WBC Count (10^3^ mm^−3^)	5.356 ± 0.741^ab^	5.320 ± 0.534^ab^	6.266 ± 0.862^bc^	7.553 ± 0.642^c^	6.606 ± 1.572^bc^	5.833 ± 1.264^bc^	4.040 ± 0.065^a^	4.070 ± 0.075^a^
Lymphocytes (%)	51.000 ± 4.582^b^	51.000 ± 6.082^b^	54.666 ± 4.163^bc^	65.333 ± 7.094^c^	65.333 ± 5.859^c^	52.333 ± 3.785^b^	45.000 ± 6.000^b^	33.333 ± 4.509^a^
Monocytes (%)	6.333 ± 0.577^ab^	6.166 ± 0.577^ab^	5.666 ± 0.763^a^	7.667 ± 0.288^ab^	6.500 ± 1.500^ab^	8.000 ± 1.322^b^	5.667 ± 1.154^a^	6.833 ± 0.763^ab^
Eosinophils (%)	0.667 ± 0.288^a^	0.833 ± 0.288^a^	1.000 ± 0.000^a^	1.667 ± 0.288^b^	0.667 ± 0.288^a^	0.833 ± 0.288^a^	0.833 ± 0.288^a^	1.000 ± 0.000^a^
Basophils (%)	0.833 ± 0.577^a^	0.833 ± 0.288^a^	1.000 ± 0.000^a^	0.833 ± 0.288^a^	0.833 ± 0.288^a^	0.667 ± 0.577^a^	0.833 ± 0.288^a^	0.167 ± 0.288^a^
Neutrophils (%)	37.500 ± 8.261^a^	36.333 ± 1.892^a^	35.667 ± 1.892^a^	30.667 ± 1.607^a^	35.167 ± 9.278^a^	37.333 ± 5.299^a^	40.833 ± 5.392^a^	54.833 ± 1.154^b^

Across each line, values with different letter superscripts are significantly different, at p < 0.05. (Waller Dunkan test). Ref: values from animals of group 1, that were not infected and received distilled water during treatment; Cipro: ciprofloxacin; Oxy: oxytetracycline.

The crude extracts and compound 1 (RSa_50_ values of 19.27 ± 4.43) presented radical scavenging activities against stable DPPH free radical in a concentration-dependent manner. The other compounds did not display appreciable radical scavenging potentials (Table 3). However, structure-activity relationship of the compounds shows that the introduction of 3-glucopyranose, C-16-OH or 3*′*-galactopyranose groups respectively in 4, 6 and 7 greatly increased the radical scavenging activities of these compounds. Microbial infections may lead to an increased formation of highly reactive molecules that can cause damage to cells and tissues (Kamlesh et al. 2007). Compound 1 (methylinositol) is a well known antioxidant product (Jiang et al. 2013). Though with significantly (p < 0.05) lower radical scavenging activity compared to L-ascorbic acid, the presence of methylinositol in the leaves of *P. pinnata* could trigger its recommendation as a natural alternative to the use of L-ascorbic acid as an antioxidant.

**Table 3:** **DPPH radical scavenging activities of the extracts and compounds from** ***P. pinnata*** **leave** ***s*** **and stems**

Test substance		RSa50 (μg/ml)
Extracts	Extract^a^	116.74 ± 4.20^cd^
	Extract^b^	99.61 ± 4.69^c^
Compounds	1	19.27 ± 4.43^b^
	2	nd
	3	nd
	4	247.30 ± 6.22^f^
	5	4763.60 ± 38.26^h^
	6	865.88 ± 26.84^g^
	7	147.23 ± 5.71^e^
Reference	Vitamin C	5.31 ± 1.09^a^

Extract^a^, leaf extract; Extract^b^, stem extract. Along each column, values with the same letter superscripts are not significantly different. Waller Dunkan (p < 0.05).; nd: not determined (RSa50 > 5000 μg/ml).

The results show that a phytmedicine may possess numerous biological properties and this is the case of *P. pinnata* with both antityphoid and radical scavenging activities. The antibacterial activity of *P. pinnata* may be enhanced by its antioxidant property since Compound 1 with a relatively good antisalmonellal activity equally displayed outstanding radical scavenging activity.

## Conclusion

The present findings support the ethno-pharmacological exploitation of *P. pinnata* in the treatment of typhoid fever and hold great perspective in the development of alternative antityphoid and antioxidant phytomedicine using Compounds 5, (3*β*)-3-*O*-(2′-Acetamido-2′-deoxy-*β*-D-glucopyranosyl) oleanolic acid, and 1, methylinositol, respectively as markers.
